# Indica rice genome assembly, annotation and mining of blast disease resistance genes

**DOI:** 10.1186/s12864-016-2523-7

**Published:** 2016-03-16

**Authors:** H. B. Mahesh, Meghana Deepak Shirke, Siddarth Singh, Anantharamanan Rajamani, Shailaja Hittalmani, Guo-Liang Wang, Malali Gowda

**Affiliations:** Genomics Laboratory, Centre for Cellular and Molecular Platforms (C-CAMP), National Centre for Biological Sciences (NCBS), Bengaluru, 560065 India; Marker Assisted Selection Laboratory, Department of Genetics and Plant Breeding, University of Agricultural Sciences, Bengaluru, 560065 India; Pacific Biosciences, Boon Lay Way, Singapore, 609964 Singapore; Department of Plant Pathology, College of Food, Agricultural and Environmental Sciences, Ohio State University, Columbus, 43210 USA; Manipal University, Manipal, 576104 India; Genomics Discovery Program, School of Conservation, Life Science and Health Sciences, TransDisciplinary University, Foundation of Revitalization of Local Health Traditions, Bengaluru, 560064 India

**Keywords:** *O. sativa*, Genome sequencing, *Indica*, Illumina, Pacific Biosciences, SSRs, SNPs, Blast resistance

## Abstract

**Background:**

Rice is a major staple food crop in the world. Over 80 % of rice cultivation area is under *indica* rice. Currently, genomic resources are lacking for *indica* as compared to *japonica* rice. In this study, we generated deep-sequencing data (Illumina and Pacific Biosciences sequencing) for one of the *indica* rice cultivars, HR-12 from India.

**Results:**

We assembled over 86 % (389 Mb) of rice genome and annotated 56,284 protein-coding genes from HR-12 genome using Illumina and PacBio sequencing. Comprehensive comparative analyses between *indica* and *japonica* subspecies genomes revealed a large number of *indica* specific variants including SSRs, SNPs and InDels. To mine disease resistance genes, we sequenced few *indica* rice cultivars that are reported to be highly resistant (Tetep and Tadukan) and susceptible (HR-12 and Co-39) against blast fungal isolates in many countries including India. Whole genome sequencing of rice genotypes revealed high rate of mutations in defense related genes (NB-ARC, LRR and PK domains) in resistant cultivars as compared to susceptible. This study has identified R-genes *Pi-ta* and *Pi54* from durable *indica* resistant cultivars; Tetep and Tadukan, which can be used in marker assisted selection in rice breeding program.

**Conclusions:**

This is the first report of whole genome sequencing approach to characterize Indian rice germplasm. The genomic resources from our work will have a greater impact in understanding global rice diversity, genetics and molecular breeding.

**Electronic supplementary material:**

The online version of this article (doi:10.1186/s12864-016-2523-7) contains supplementary material, which is available to authorized users.

## Background

Rice (*Oryza sativa L.*) is a staple food for more than half of the world’s population. India is believed to be a center of origin and diversity of rice. India is the second most rice producer in the world and 65 % of its population depends on rice as a staple food. Rice is a model cereal crop with small genome size, short generation time, diploid (2n = 24) and amenable for genetic manipulation. Due to its global importance, several genomes of rice cultigen including *japonica* (Nipponbare [1]*), indica* (93-11 [2] and IR64 [3]) and *aus* (Kasalath [4]) have been sequenced. Currently gold standard assembly and annotation are available for *japonica* rice. Although over 80 % of rice cultivation in the world under *indica* rice cultivation however, genomic resources are lacking for this subspecies. Due to non-availability of proper genome assembly, *indica* genome studies are still using Nipponbare genome as a reference. This will introduce the potential bias in analysis and may not capture conclusive results at the nucleotide and chromosomal level for *indica* subspecies [3]. Thus, we and other researchers [3] believe that creating an *indica* reference rice genome is essential for genome-wide studies, which will enable genome assisted *indica* rice breeding program.

Here we report the *de novo* genome assembly and annotation of *indica* cultivar, HR-12. This cultivar was bred and released in India (http://www.drricar.org). It has good agronomic traits, but highly susceptible to blast disease [5], caused by *Magnaporthe oryzae*. Rice blast is a serious constraint in rice production and utilization of resistant (R) genes in variety development has become the most effective method of blast disease management. Sequencing of highly resistant and susceptible varieties will enable the identification of novel R genes and their deployment in rice breeding programme. In addition to HR-12, we resequenced whole genomes of three *indica* cultivars, which are highly susceptible (Co-39) and resistant (Tetep and Tadukan) to rice blast. Our *indica* rice sequencing efforts have complemented the global rice genomic resources, which eventually will help to characterize *indica* rice germplasm to identify genes for agronomically important traits including disease, pest and yield attributing traits.

## Methods

### Indica rice cultivars and genome size estimation

The HR-12 (Himmatsagar Rice-12) was derived from Raja Hansa (http://inger.irri.org). Subsequently HR-12 was used to develop Hamsa (HR-12 x TN-1) and Tellahamsa (HR-12 x TN-1) rice varieties (Additional file 1). The Co-39 variety was developed by crossing Culture340 and Kannagi. These two varieties have been widely used as susceptible checks in rice blast screening nursery. Tetep and Tadukan are used as resistance checks, and donor parents for blast resistance breeding, however the genealogy information is not available. The seeds of HR-12, Co-39, Tetep and Tadukan were sown in a PVC pot containing red earth and fertilizers. Twenty one days old leaves from Co-39, HR-12, Tetep and Tadukan were collected and chopped into pieces in nuclear isolation buffer (Hypotonic Propidium Iodide, 50 μg/mL in 3 g/L TriSodium citrate Dihydride containing 0.05 % (v/v) of Nonidet P-40 containing 2 mg/mL RNase A) and samples were processed as per the protocol suggested by Krishna [6]. Debris was filtered and stained nuclei were analysed using BD FACS at Central Imaging and Flow Cytometry Facility (CIFF), C-CAMP, NCBS, Bengaluru, India. Values of rice nuclear DNA was estimated by comparing rice nuclear peak on the linear scale with the peak for *Pisum sativum* included as an internal standard.

### Nucleic acid isolation

Genomic DNA was isolated from four (HR-12, Co-39, Tetep and Tadukan) varieties as per the manufacturer’s instruction (DNAeasy Plant Mini Kit, Cat # 69104, USA). DNA quality was assessed by Nanodrop and DNA was quantified using Qubit (Applied Biosystems).

### Paired-end (PE) and matepair (MP) library preparation, and Illumina sequencing

One micro gram of genomic DNA was fragmented in the range of 300 to 400 bases using ultra Sonicator (S220, Covaris, USA). Then PE library of HR-12, Co-39, Tetep and Tadukan samples were prepared using Tru Seq DNA sample preparation kit v2 (Catalog No: FC-121-2001, Illumina) as per the manufacture’s instruction. PE libraries were sequenced using Illumina HiSeq1000 and the length of sequence was 101 nts from both ends of the fragment. The MP library (insert size upto 12Kb) was prepared only for HR-12 sample by Nextera MP sample preparation kit (catalog No.: FC-132-1001, Illumina) and sequenced 2×51 nts by Illumina HiSeq1000.

### Strand-specific RNA seq library preparation and sequencing

Total RNA was isolated from HR-12 leaves using Direct-zol RNA MiniPrep kit (Catalog No. R2050, Zymo Research) and RNA integrity and quantity was assessed by Bioanalyzer using Agilent RNA 6000 nano chip. TruSeq stranded total RNA library preparation kit v2 (Catalog No.: RS-122-2201) from Illumina was used to prepare strand-specific RNA sequencing (ssRNA-seq) library by following manufacture’s instruction. The ssRNA-seq library was sequenced 2x101 nts by Illumina HiSeq1000.

### PacBio library preparation and sequencing

Around 20 micrograms of high quality genomic DNA was sheared using Hydroshear. The Bluepippin was used to select 20Kb double-stranded DNA fragments. Then, DNA fragments were end repaired and ligated with universal hairpin adapters. Subsequent steps were followed as per the manufacture’s instruction to prepare SMRTbell library. The library was sequenced in PacBio RS SMRT instrument.

### Illumina data output and data preprocessing

The low quality bases (quality less than Q30 or the accuracy less than 99.99 % of the base called) and adapter sequence contaminations in raw reads of Illumina sequencing (PE, MP and ssRNA-seq) was processed using FASTX-Toolkit (http://hannonlab.cshl.edu/fastx_toolkit/index.html).

### Short read (Illumina) *de novo* genome assembly of HR-12

The quality processed Illumina reads (PE and MP reads) were used for *de novo* assembly using three *de novo* genome assemblers viz., Velvet [7], SOAPDenovo2 [8] and MaSuRCA [9]. The quality of genome assemblies were assessed using QUAST [10].

### Gap filling of short read assembly using PacBio long reads, contigs scaffolding and anchoring

Raw PacBio reads were used for gap filling and to upgrade short read assembly using PBJelly pipeline [11]. Gap filled assembly was further scaffolded by L_RNA_scaffolder [12] using transcripts assembled by Trinity [13]. The pseudomolecules of HR-12 were constructed by anchoring HR-12 contigs on to pseudomolecules of Nipponbare genome (version 7.0) with ABACAS [14]. The synteny map of rice genomes was generated with default parameters by SyMAP [15]. The genome completeness of short read and gap filled assemblies were checked by CEGMA [16].

### Gene prediction and functional annotation of HR-12 genome

The HR-12 pseudomolecules were subjected for gene prediction with MAKER-P [17] version 2.31.6 by providing expressed sequences (trinity assembled HR-12 transcripts, ESTs, cDNA and mRNA) of *Oryza* (NCBI). Protein domain structures and gene ontology (GO) terms were assigned using InterProScan5 software [18]. Functional annotation of genes was done by searching homology against rice protein sequences of SwissProt (http://www.uniprot.org) using BLASTp alignments with an e-value threshold of 1^e-10^. The synonymous (Ks) and non-synonymous (Ka) substitution rate was calculated using NG [19] method in Ka/Ks calculator [20].

### Annotation of HR-12 specific genes

Nipponbare (version 7.0) and 93–11 gene models were downloaded from MSU rice genome FTP site (ftp://ftp.plantbiology.msu.edu/pub/data/Eukaryotic_Projects/o_sativa/annotation_dbs/pseudomolecules/version_7.0/all.dir/) and BGI (http://rice.genomics.org.cn/rice/index2.jsp) site, respectively. The homology search was carried out with BLASTp using Nipponbare and 93–11 proteins as subject and HR-12 proteins as a query; the e-value cutoff was set to 1^e-10^.

### Repeat identification and prediction of Simple Sequence Repeats (SSRs)

*De novo* repeat prediction was performed using Repeat Masker 4.0.5 (http://www.repeatmasker.org) using *Oryza sativa* repeat library in Repbase as a reference. The SSRs were predicted using Microsatellite Identification tool [21]. Rice SSR markers in Gramene website (http://archive.gramene.org/markers/microsat/all-ssr.tab) were used for performing an electronic PCR [22] (e-PCR) to check polymorphic and novel SSRs in HR-12 genome.

### Identification of SNPs, InDels and functional annotation of variants

We used Illumina data of HR-12, Co-39, Tetep and Tadukan for variant analysis. The high quality (Q30) Illumina reads were mapped to Nipponbare genome using Burrows-Wheeler Aligner (BWA) V0.7.9a [23]. Alignments with mapping quality (‘q’ option in samtools) <60 (Phred-scaled) were filtered using SAMtools [24]. Duplicate reads were removed using Picard tool v1.115 (http://broadinstitute.github.io/picard/). We performed local realignment around InDels to correct mapping related artifacts using InDelRealigner tool in GATK V3.3-0. Then base quality recalibration was performed using BaseRecalibrator tool in GATK V3.3-0. Variant calling was performed with a minimum Phred-scaled confidence threshold of 30, and a minimum Phred-scaled confidence threshold for emitting variants at 10 using HaplotypeCaller in GATK V3.3-0 across all four samples [25, 26]. Functional annotation and genetic consequences (effect on gene) of common variants (SNPs and InDels) of susceptible and resistant varieties was annotated using SnpEff tool V4.1b [27]. The Nipponbare genome version 7.0 was used as a reference to annotate SNPs and InDels.

### Mining of blast disease resistance genes in *indica* cultivars

The sequences of twenty-two cloned blast *R*-genes (NCBI) were subjected to BLASTp alignment (e-value cutoff 1^e-10^, minimum 70 % identity and query coverage) with proteins sequences of HR-12, Co-39, IR64 (http://schatzlab.cshl.edu/data/rice/), Tetep, and Tadukan.

## Results and discussion

### *De novo* short read HR-12 genome assembly

The genome of *indica* rice cultivar, HR-12 was assembled using combination of short reads (PE and MP libraries) from Illumina and long reads (Additional file 2) from Pacific Biosciences (Additional file 3). Initially, the Illumina short reads were assembled using three *de novo* assemblers Velvet, SOAPdenovo2 and MaSuRCA. Among three assemblers, MaSuRCA covered highest genome size of 340.12 Mb and it had least number of N’s as compared to other two assemblers. All three assemblers resulted comparatively lower contig N50 (Additional file 4), which might result due to inherent disadvantages of short read assemblies, such as poor repeat resolution [3, 28], missing exons and genes and genes split between scaffolds [29]. Less number of contigs in Velvet [7] and SOAPDenovo2 [8] assemblies were because of higher number of ‘N’s in the assembly, which were used to merge adjacent contigs into scaffolds (Additional file 4). Based on quality assessment by QUAST, MaSuRCA assembly was chosen for further analysis since it showed more improvement with respect to genome coverage (74.80 %), lower number of gaps (47479) and higher number of genes covered (48428) (Additional file 5).

### Improvement of short read *indica* rice genome assembly using PacBio long reads

**Fig. 1:**
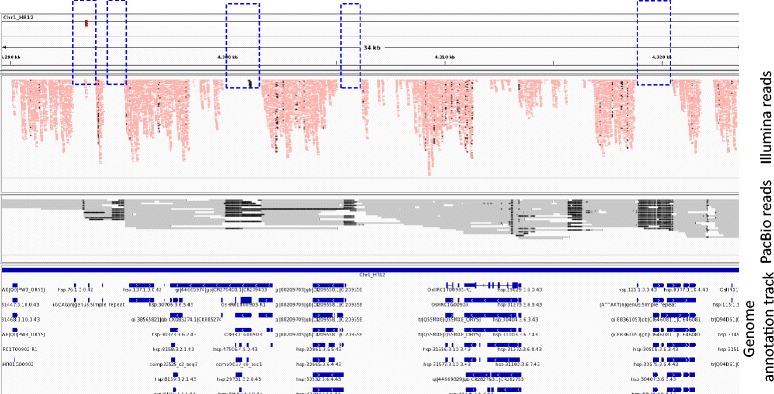
Diagram depicting the assembly gaps closed by PacBio long reads. Gaps in short read assembly are shown in broken rectangles and gene models are represented in genome annotation track

To improve assembly quality and overcome the limitations of short read assembly, HR-12 genome was sequenced using PacBio RS SMRT platform with 20x coverage. The PacBio reads were used to fill the gaps in the short read assembly (Fig. 1) which improved HR-12 genome with respect to genome size, N50 and number of contigs/scaffolds. The genome assembly size was increased by 49.52 Mb which accounts for over 86 % of estimated HR-12 genome (454 Mb). The average genome size of four *indica* cultivars was found to be higher (457 Mb) as compared to previous report [30] (Additional files 6 and 7). The N50 of gap filled assembly was increased from 6.82 to 26.46 Kb. Similarly, the number of scaffolds was reduced from 98939 to 61001 (Additional file 4). The Core Eukaryotic Genes (CEG) mapping approach has resulted 82.26 % and 94.35 % genes in short read and gap filled assemblies, respectively. According to our data, the gap filled *indica* rice, HR-12 genome assembly is much better with respect to genome size, number of genes, scaffold N50 and genome completeness as compared to other rice genomes including *indica* (93–11 and IR64), *aus* (Kasalath) and *japonica* (Nipponbare) (Table 1). The overall assembly quality comparison with other published genomes reiterated the importance of PacBio long reads in generating gold standard genomes for complex eukaryotic organisms. In a recent study, reference mapping of sequence reads of 50 rice accessions to *japonica* (Nipponbare), *indica* (93–11) and *aus* (Kasalath) genomes showed higher rate of mapping to Kasalath followed by 93–11 and Nipponbare genomes [4]. This indicates that 93–11 genome is not well assembled and annotated [3, 4].

**Table 1:** Comparison of whole genome sequencing of *Oryza sativa* subspecies

Details	HR-12 (current study)	93–11 [2]	IR-64 [3]	Kasalath [4]	Nipponbare [1]
Subspecies	*indica*	*indica*	*indica*	*aus*	*japonica*
Sequence depth	73x (Illumina) and 20x (PacBio)	4.2x (Sanger) and 36x (Illumina)	110x (Illumina)	6x (Roche 454) and 148X (Illumina)	6x (Sanger), 62x (Illumina) and 2.57x (Roche 454)
Sequencing technologies	Illumina and PacBio	Sanger and Illumina	Illumina	Illumina and Roche 454	Sanger, Illumina and Roche 454
Genome assembler(s)	MaSuRCA and PBJelly	RePS and SOAPDenovo	ALLPATHS-LG	Celera Assembler and String Graph Assembler	TIGR Assembler
Genome size (Mb)	389.77 (389.60)	374.55 (359.41)	345.9 (321.2)	401.14 (328.25)	374.47 (374.31)
No. of N's (Mb)	0.17	15.14	24.7	72.89	0.17
Total no. of contigs/scaffolds	59,692	50,231	26,160	51,550	NA
a. Anchored contigs/scaffolds	43,781	35,415	NA	36,932	NA
b. Unanchored contigs/scaffolds	15,911	14,816	NA	14,618	NA
N50 (Kb)	28.5	6.69	22.2	13.73	NA
No. of genes	56,284	40,464	37,758	53,662	55,986
CEG mapping (%)	94.35	95.97	94.35	95.97	95.97

Value in parenthesis indicate genome size without ‘N’ content

*NA* Not available, *No.* Number, *Mb* Mega bases, *Kb* Kilo bases

*N50* 50 % of the contigs represent this contig length

*CEG* Core Eukaryotic Genes

### HR-12 contigs ordering on Nipponbare chromosomes

Ordering and orienting of contigs/scaffolds onto pseudomolecules has facilitated identification of gaps, closure of gaps and also comparative whole genome analyses. We scaffolded HR-12 contigs by utilizing stranded RNA sequencing data, which reduced scaffold numbers from 61001 to 59692. Over 73 % of HR-12 contigs (43781 out of 59692 contigs) anchored onto pseudomolecules of Nipponbare with 80 % sequence identity and remaining 15911 were unanchored. The unanchored contigs could be part of structural variants like insertions, inversions and translocations. The minimum and maximum contig length of unanchored contigs was 226 and 392500 bp, respectively. The N50 was 36469 bp with an average contigs length of 6642 bp. Nearly 55.40 % (8815) and 60.34 % (9600) of HR-12 contigs aligned to Nipponbare and 93–11 genomes with 100 % query coverage and 80 % sequence similarity, which confirmed that unaligned contigs were part of rice genome. Around 1.55 % (247) of contigs did not show any alignment to Nipponbare genome indicating their absence in the reference genome.

The synteny map of HR-12, Nipponbare and 93–11 (Additional file 8) showed that most of the genomic blocks conserved across all three genomes with few translocations. Around 79 % and 74 % of HR-12 genome is in syntenic with Nipponbare and 93–11 genomes, respectively which restate that 93–11 genome is not well assembled.

### Gene prediction and functional annotation

The gene prediction using MAKER-P in gap filled assembly of HR-12 genome yielded 56,284 protein-coding transcripts. We performed two-way comparison of genes from short read and gap filled assemblies, resulting into 31,933 genes with 100 % identity. In addition, 2615 genes were annotated which were unique to gap filled assembly but absent in short read assembly. About 38.6 % (21,736) of genes were fragmented in short read assembly as compared to gap filled assembly. This indicates that use of PacBio reads has significantly improved the *indica* rice genome annotation.

Genes of gap filled assembly were also compared with genes of Nipponbare and 93–11. There were 54,849 genes (e-value of 1^e-10^) commonly present in both HR-12 and Nipponbare. Similarly 54,130 genes were commonly present in HR-12 and 93–11. This indicates that over ~97 % of annotated rice genes from *indica* (93–11) and *japonica* (Nipponbare) were annotated in HR-12 gap filled assembly. Overall, 1950 genes were unique to HR-12 genome in comparison with Nipponbare and 93–11 genomes (Additional file 9). These unique genes in HR-12 were annotated and classified as proteins of unknown function (96), uncharacterized proteins (1349) and proteins with known function (475). There were 30 resistance genes with NBS-LRR, LRR and kinase domains (Additional file 9).

### Ka/Ks analysis for annotated proteins

To determine the evolutionary selection pressure on proteome between *indica* (HR-12 and 93–11) and *japonica* (Nipponbare) genomes, synonymous amino acids substitution rates (Ks) and non-synonymous amino acids substitution rates (Ka) were calculated. The Ka/Ks ratio can reflect the selection pressure between gene pairs (homologs) caused by evolutionary forces like natural mutations. This homolog group analyses resulted 3424 and 5527 gene pairs between *indica-indica* (HR-12 v/s 93–11), and *indica-japonica* (HR-12 v/s Nipponbare), respectively. Homolog groups were classified into three categories based on Ka/Ks ratio with probability value of <0.05 (Fisher exact test). The *indica-indica* homologs comparison yielded 32 and 3392 genes being positively and negatively selected with probability value of <0.05 (Fisher exact test), respectively. Similarly, *indica-japonica* homologs comparison showed 29 and 5498 genes were under positive (Ka/Ks ratio >1) and negative (Ka/Ks ratio <1) selection, respectively (Additional file 10).

### Genome-wide comparison of SSRs and SNPs in sequenced genomes

**Fig. 2:**
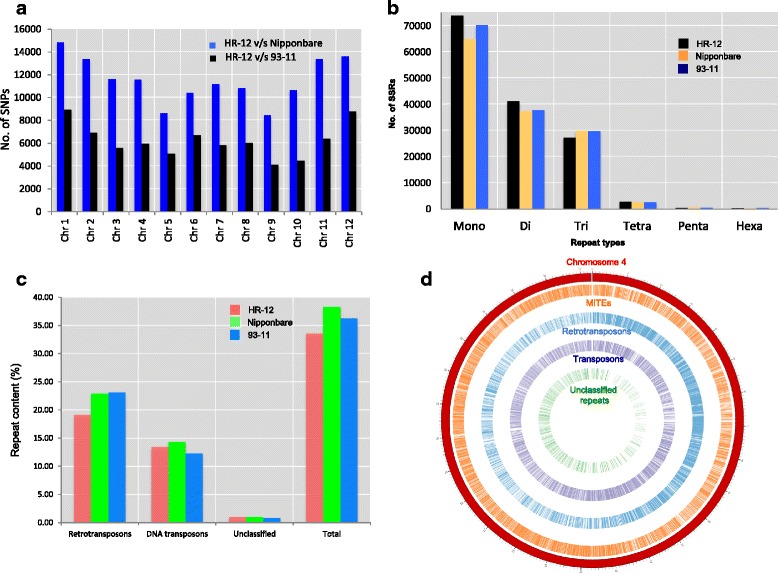
Genome-wide comparison of HR-12, 93–11 and Nipponbare genomes for SNPs, SSRs and repeats. **a** Number of SNPs, (**b**) number of SSRs, (**c**) distribution of repeat elements and (**d**) density of repeats on chromosome 4 of HR-12 genome

The SSRs are repetitive DNA sequences used as co-dominant molecular markers to determine genetic diversity and mapping of genes/QTLs. Identification of SSRs in genome sequences will increase the availability of more genomic resources. In total, 114508, 145371, 135501 and 141177 SSRs were identified in Illumina HR-12 assembly, long read HR-12 assembly, Nipponbare and 93–11 genomes, respectively (Fig. 2b and Additional file 11). Mono and di-nucleotide repeats were more in HR-12 genome as compared to Nipponbare and 93–11. The ‘AT’ (di-repeats) and AAG, AGG (tri-repeats) repeats were more predominant in HR-12 genome (Additional file 11). The ‘AT’ rich di-nucleotide repeats are reported to be most abundant in rice genome as compared to other SSRs [31]. Among tetra- repeats; AAAC, AAAG, AAAT, AACC, AACG, AAGG, ACAT, ACGC, ACGG, ACGT and AGAT types were more abundant in HR-12 genome (Additional file 11) as compared to Nipponbare and 93–11 genomes.

More than 19000 SSRs are available in the Gramene database (http://archive.gramene.org/markers/microsat/all-ssr.tab). These sequences were used for e-PCR analysis in HR-12, Nipponbare and 93–11 genomes. There were 11296 (58.55), 16144 (83.68) and 13429 (69.61) SSRs mapped to HR-12, Nipponbare and 93–11 genomes, respectively. This indicates that publicly available SSR markers were skewed towards *japonica* genome. Among 11296 SSRs from HR-12 assembly, 1095 SSRs were monomorphic and 10201 SSRs were polymorphic across three genomes. The pair-wise comparison of polymorphic SSRs has yielded 8914 polymorphic SSRs between HR-12 and Nipponbare, and 8445 SSRs polymorphic between HR-12 and 93–11. As expected, SSRs were less polymorphic within *indica* rice (HR-12 and 93–11) (*indica* type) and highly polymorphic between *japonica* rice (Nipponbare). Among 11296 SSRs in HR-12, 1617 SSRs were unique to HR-12 genome (Additional file 12). These unique SSRs (1617) were compared with sequenced genomes of other *indica* varieties, Tetep and Tadukan. Out of these, 1260 SSRs were found in both Tetep and Tadukan, which resulted 209 SSR markers unique to HR-12 genome. Out of remaining 148 SSRs, 128 SSRs were common between HR-12 and Tetep but absent in Tadukan genome and 20 were common between HR-12 and Tadukan.

The comparison of Gramene SSRs in HR-12 (11296) and predicted SSRs (56560) revealed that all 11296 public SSRs were present in predicted SSRs. Elimination of publicly available SSRs in predicted SSRs of HR-12 yielded 47378 SSRs to be novel from *in silico* prediction for *indica* genome. Among these, 20547 di, 18760 tri, 1040 tetra, 332 penta and 6699 were complex SSRs. The conserved flanking regions (100 bp upstream and downstream from SSR motif) of 43834 and 44123 SSRs of HR-12 (47378) were mapped to Nipponbare and 93–11 genomes, respectively. Whereas 3533, 3255 HR-12 SSRs were unique to HR-12 and did not map to Nipponbare and 93–11 genomes.

Genome-wide comparison of SNPs between HR-12 and 93–11, HR-12 and Nipponbare discerned higher level of polymorphism between *indica-japonica* comparison (Fig. 2a). These species-specific variations can be used for marker assisted breeding, positional cloning and evolutionary studies.

### Repeats in HR-12 genome

Repetitive DNA sequence can account for substantial portion of the many eukaryotic genomes and lead into genome expansion, gene disruption and gene duplication. Total 34.9 % repeats were found in HR-12 genome (gap filled assembly) as compared to Nipponbare (39.64 %) and 93–11 (37.62 %) (Fig. 2c). Total interspersed repeats were the major elements (33.57 %), consisting of retro-elements (19.14 %), DNA transposons (13.44 %) and unclassified repeats (1 %). In case of retro-elements, long terminal repeat (LTR) had a highest fraction (17.72 %), followed by LINEs (0.98 %) and SINEs (0.43 %). In case of DNA transposons, tourist/harbinger elements content was more (2.84 %), followed by Tc1-IS630-Pogo (2.61 %), hobo-Activator (0.52 %), En-Spm (0.43 %) and MuDR-IS905 (0.14 %). Similar trend of repeat distribution was observed in case of Nipponbare and 93–11 genomes (Additional file 13). Chromosome-wise repeat distribution (Additional file 14) displayed highest repeat content of 39.60 % on chromosome 4 and the lowest of 31.74 % on chromosome 3. The overall distribution of retro-transposons, DNA transposons and Miniature Inverted-repeat Transposable Elements (MITEs) in HR-12 genome for chromosome 4 is represented in Fig. 2d.

### Whole genome sequencing of blast susceptible and resistant *indica* rice varieties

**Fig. 3:**
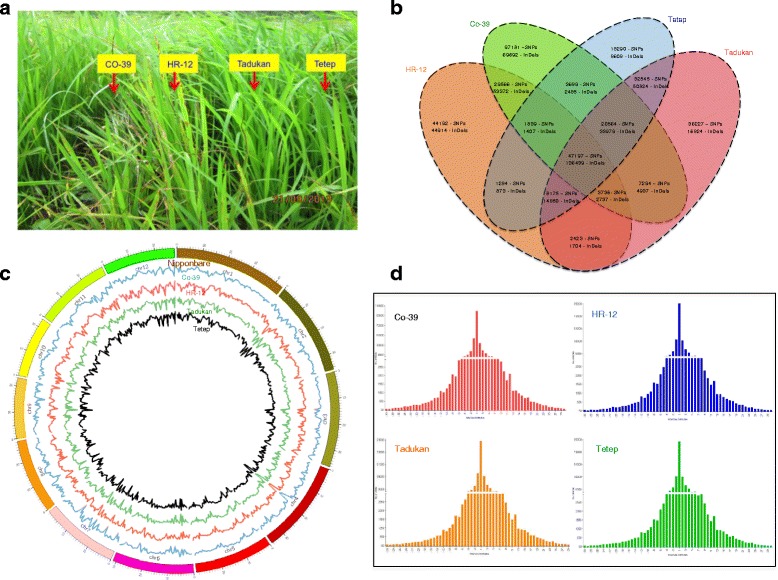
Genome-wide variants identified in HR-12, Co-39, Tadukan and Tetep *indica* cultivars. **a** Blast symptoms, (**b**) Venn diagram showing unique and shared SNPs and InDels between and among susceptible and resistant varieties, **c**) Genome-wide distribution of SNPs in Co-39, HR-12, Tadukan and Tetep and **d**) InDels distribution in Co-39, HR-12, Tadukan and Tetep

We sequenced *indica* cultivars, which are highly resistant (Tetep and Tadukan) and susceptible (HR-12 and Co-39) to rice blast disease caused by *Ascomycetes* pathogen *Magnaporthe oryzae* (Fig. 3a). Mapping of sequence reads to Nipponbare genome enabled us to identify large number of variants in Co-39 (211066 SNPs and 305048 InDels), HR-12 (138412 SNPs and 256296 InDels), Tadukan (158161 SNPs and 262294 InDels) and Tetep (133583 SNPs and 383048 InDels) (Fig. 3c). Large fraction of SNPs and InDels were found in upstream and downstream regions of annotated genes (Additional file 15). The transition to transversion (Ts/Tv) ratio was 1.69, 1.58, 1.61 and 1.58 in Co-39, HR-12, Tetep and Tadukan, respectively. The SNPs were located at the intervals of 1764, 2692, 2791 and 2357 nts in the genomes of Co-39, HR-12, Tetep and Tadukan, respectively. Similarly, the InDel rate was per 1207, 1449, 1493 and 1419 bases in Co-39, HR-12, Tetep and Tadukan, respectively. One base insertions (+1) and deletions (−1) were more [32] as compared to other types of InDels (Fig. 3d).

Among blast disease susceptible varieties (HR-12 and Co-39), 29566 SNPs and 53372 InDels and among resistant varieties (Tetep and Tadukan), 32545 SNPs and 50824 InDels were common (Fig. 3b). In blast susceptible varieties, 44192 SNPs and 44914 InDels were unique to HR-12 and 97181 SNPs and 69692 InDels were unique to Co-39. Similarly in blast resistant varieties, 36227 SNPs and 16824 InDels were unique to Tadukan where as 18290 SNPs and 8608 InDels were unique to Tetep.

### Functional annotation of variants in blast resistant and susceptible varieties

Commonly occurring variants (29566 SNPs and 53372 InDels in susceptible; 32545 SNPs and 50824 InDels in resistant varieties) were annotated to know their functionality in the genome (Additional file 16). In total, 24431 and 27562 genes were mutated (SNPs and InDels) in susceptible and resistant varieties, respectively. We further classified these genes based on their protein domains and focused on genes involved in host defense mechanism (resistance genes) such as protein kinase (PK), nucleotide-binding adapter shared by APAF-1, R-proteins and CED-4 (NB-ARC), and leucine-rich repeats (LRR) domains. We observed defense related genes have accumulated more mutations (non-synonymous) in resistant varieties as compared to susceptible varieties (Table 2). There were 40 NB-ARC, 20 LRR, 93 PK genes were mutated (SNPs and InDels) in resistant varieties. In susceptible varieties, 16 NB-ARC, 17 LRR and 67 PK genes were mutated. This functional annotation of variants is highly useful to develop PCR-based functional markers [33] to screen large set of rice germplasm and identify novel alleles of R genes, enabling breeders to rapid introgression of resistance genes and gene pyramiding [34] to elite cultivars for durable blast resistance.

**Table 2:** Mutation in defense related genes in *indica* rice varieties

Domain Name	Domain ID	No. of genes mutated			
		Blast resistant varieties (Tetep and Tadukan)		Blast susceptible varieties (HR-12 and Co-39)	
		SNPs	InDels	SNPs	InDels
NB-ARC	PF00931	15	25	6	10
LRR	PF00560, PF08263, PF12799	3	17	2	15
Protein Kinase	PF00069, PF07714, PF13947, PF03727, PF00406, PF00781, PF00485	31	62	17	50
TOTAL		49	104	25	75

*NB-ARC* Nucleotide-binding adaptor shared by APAF-1, R proteins, and CED-4

*LRR* Leucine Rich Repeats

### Mining of blast disease resistant genes in *indica* rice varieties

**Fig. 4:**
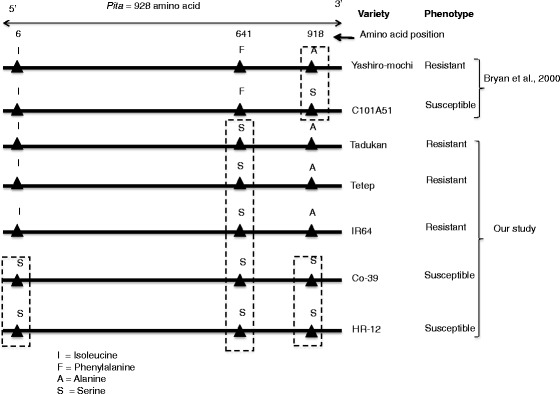
Structure of *Pi-ta* gene in *indica* cultivars as compared to reference gene

Till-date, 22 blast resistant *R*-genes cloned [35–39] from several rice varieties, which confers resistance against *Magnaporthe* isolates. To assess the spectrum of R-genes in resistant (Tetep, Tadukan and IR64) and susceptible (Co-39 and HR-12) cultivars, we performed protein-protein alignment of R-genes. The R-genes such as *Pi37*, *Pid2, Pid3, Pi25, Pish and Pi64* were conserved at structure level in all *indica* varieties, however, several SNPs and InDels were interrupted these genes. The *Pi54* (*Pikh*) gene was present in Tadukan and Tetep but absent in Co-39, HR-12 and IR64. *Pi-ta*, a broad spectrum resistant gene was found in all four *indica* varieties. However, single amino acid substitution (Ala to Ser) was observed at 918 in *Pi-ta* protein, which reported to be responsible for determining resistance specificity [40]. We observed similar Ser substitution in *Pi-ta* protein in blast susceptible varieties (Co-39 and HR-12). In addition, we identified novel amino acid (Phe to Ser) substitution at 641 residues in all *indica (*Co-39, HR-12, IR64, Tadukan, Tetep) varieties (Fig. 4). Other genes such as *Pib, Piz-t, Pik-m, Pi5, pi21, Pb1, Pik, Pik-p and Pi1* were absent in all *indica* varieties (Table 3). Majority of R-genes were either mutated or fragmented in case of susceptible varieties. The R genes in host and AVR genes in pathogen follow gene -for -gene hypothesis [41]. Survey of avirulent genes (AVR) in *Magnaporthe* population isolated from HR-12 from Southern India showed predominance of *AVR-Pizt*, *AVR-Pita*, *AVR-Pik*, *AVR-Pii*, and *AVR-Pia* (Unpublished). The cognate R-genes like *Piz-t*, *Pi-ta*, *Pik* and *Pia* were absent in HR-12. Similarly, *Magnaporthe* isolates from Co-39 showed presence of *AVR-Pizt*, *AVR-Pii* and *AVR-Pik* and the cognate R-genes, *Piz-t* and *Pik* genes were absent in Co-39 genome. Thus, study of R and AVR genes in the crop ecosystems play an important role to understand the evolution of new pathotypes and to design better plant breeding strategies.

**Table 3:** Distribution of cloned blast resistance genes in sequenced rice varieties

‘R' gene	Donor Variety	Group	HR-12	Co-39	Tetep	Tadukan	IR-64
*Pib*	Tohoku IL-9	*Japonica*	-	-	-	-	M
*Pi-ta*	Yashiro-mochi	*Japonica*	-	-	+	+	+
*Pi54 (Pik-h)*	Tetep	*Indica*	-	-	+	+	-
*Pid2*	Digu	*Indica*	M	M	M	M	M
*Pi9*	75-1-127	*Indica*	M	-	-	-	M
*Piz-t*	Toride 1	*Japonica*	-	-	-	-	-
*Pi37*	St.No. 1	*Japonica*	M	M	M	M	M
*Pi36*	Kasalath	*Indica*	M	-	-	M	M
*Pik-m-TS1*	Tsuyuake	*Japonica*	-	-	-	-	-
*Pik-m-TS2*	Tsuyuake	*Japonica*	-	-	-	-	M
*pi21*	Owarihatamochi	*Japonica*	-	-	-	-	-
*Pit*	Nipponbare	*Japonica*	M	-	M	-	M
*Pi5-1*	RIL260	*Japonica*	-	-	-	-	M
*Pi5-2*	RIL260	*Japonica*	-	+	-	-	-
*Pid3*	Digu	*Indica*	M	M	M	M	M
*Pb1*	St.No. 1	*Japonica*	-	-	-	-	-
*Pish*	Shin 2, Norin 22	*Japonica*	M	M	M	M	M
*Pi25*	Gumei 2	*Japonica*	M	M	M	M	M
*Pia (RGA4)*	Sasanishiki, Aichi-asahi	*Japonica*	-	M	M	M	M
*Pik-p-1*	K60	*Japonica*	-	-	-	-	M
*Pik-p-2*	K60	*Japonica*	-	-	-	-	M
*Pik-1*	Kusabue	*Japonica*	-	-	-	-	M
*Pik-2*	Kusabue	*Japonica*	-	-	-	-	M
*Pi54rh*	*O. rhizomatis*	Wild species	-	-	M	M	-
*Pi1-5*	LAC23, C101LAC	*Indica*	-	-	-	-	M
*Pi1-6*	LAC23, C101LAC	*Indica*	-	-	-	-	M
*Pi64*	Yangmaogu (YMG)	*Japonica*	M	M	M	M	M

+ = Present

- = Absent

*M* Mutated but protein structure retained

## Conclusions

High quality genomic resources are prerequisite for rice breeding. The available *indica* reference genomes 93–11 and IR64 were sequenced and assembled using short read sequencing technology. Inherent drawbacks of short read genome assemblies may cause alignment problem during reference mapping and study of structural variations. Due to non-availability of high quality *indica* reference genome, Illumina reads from 3000 rice accessions (*indica*, *aus*, *tropical japonica*, *temperate japonica* and *aromatic*) were mapped to Nipponbare genome to identify variants [42]. Although another *indica* genome IR64 [3] is sequenced, its chromosomes consist of complex recombination of fragments (mosaic) from the genealogy of more than 38 parents [43] including *indica*, *japonica* and wild species. With advent of third generation sequencing technology, it is possible to sequence plant genomes with higher accuracy and coverage. Thus, we report improved *de novo* assembly of *indica* cultivar HR-12 using combinatorial approach of short and long reads which covers over 86 % of estimated genome size. The gap filling strategy with the help of long reads has improved the short read assembly with respect to genome size, repeat content, and number of protein coding genes. Whole genome comparison of HR-12, 93–11 and Nipponbare genomes revealed 1950 genes and 1617 SSRs unique to HR-12 genome with similar level of repeat content.

Rice blast is a major biotic stress in rice, which reduces yield significantly. To understand disease resistance genes, we sequenced highly resistant (Tetep and Tadukan) and susceptible (Co-39, HR-12) *indica* cultivars. Functional annotation of SNPs and InDels in *indica* cultivars showed higher non-synonymous substitutions in defense related genes [44, 45] containing NB-ARC, LRR and PK domains among resistant varieties indicating strong diversifying selection to confer resistance to fast evolving blast pathogen. Allele mining for resistance genes in all sequenced genomes showed presence/absence polymorphism and large number of structural variations. Most of the R genes were conserved in resistant cultivars with point mutations and InDels whereas loss of R-gene structure was noticed in susceptible cultivars. The broad-spectrum resistance in Tadukan, Tetep and IR64 could be attributed to presence of intact *Pi-ta* and *Pi54* (absent in IR64*)*. The rice cultivars, Tadukan and Tetep were found to be resistant against most of blast races across the globe [46, 47] and these have been used in IRRI for developing IR64. Previously, high rate of mutation in R genes have been reported in Rice (*Xa21*) [48] and Maize *(Rp1)* [49]. These allelic variations created by mutations will result in evolution of novel R genes/alleles and selection of genes that can recognize pathogen avirulence gene products. Identification of R genes/alleles is a prerequisite for effective utilization of genetic and genomic resources in modern plant breeding, which is driven by new genomics tools.

### Data availability

The raw sequence reads deposited under NCBI Sequence Read Archive (SRA) accession numbers SRP067809 (HR-12), SRP067775 (Co-39), SRP067810 (Tetep), and SRP067808 (Tadukan). The whole genome shotgun projects have been deposited at DDBJ/EMBL/Genbank under the accessions AZTA02000000 (HR-12), LQHE01000000 (Co-39), LQHG01000000 (Tetep), and LQHF01000000 (Tadukan).

## Additional files

Additional file 1: Genealogy of HR-12 variety. (PPTX 76 kb) Additional file 2: Sequence data size for rice cultivars. (XLSX 15 kb) Additional file 3: Analysis workflow followed for assembling Illumina and PacBio sequence reads of HR-12. (PPTX 129 kb) Additional file 4: Short and long read assembly statistics of HR-12. (XLSX 16 kb) Additional file 5: Assembly quality assessment by QUAST. (XLSX 16 kb) Additional file 6: Genome sizes of *indica* cultivars. (XLSX 16 kb) Additional file 7: Frequency histograms of numbers of nuclei per channel as a function of relative fluorescence in *Pisum* (internal standard), Co-39, HR-12, Tetep and Tadukan. The ‘x’ and ‘y’ axes represents number of nuclei and linear fluorescence, respectively. (PPTX 298 kb) Additional file 8: Comparison of syntenic blocks among HR-12, 93–11, and Nipponbare genomes. (a) syntenic blocks between HR-12 and 93–11. (b) syntenic blocks between HR-12 and Nipponbare and (c) syntenic blocks between HR-12, 93–11 and Nipponbare. (PPTX 3804 kb) Additional file 9: Unique genes in HR-12 genome. (XLSX 97 kb) Additional file 10: Gene under positive selection in *indica-indica* and *indica-japonica* comparisons. (XLSX 21 kb) Additional file 11: Number of SSRs in Illumina short and PacBio long read assemblies (a), Number of tri and tetra SSRs of HR-12, 93–11 and Nipponbare genomes (b). Distribution of tetra type SSRs in HR-12, Nipponbare and 93–11 genomes (c). (PPTX 183 kb) Additional file 12: Unique SSRs in HR-12 genome in comparison with Nipponbare and 93–11. (XLSX 133 kb) Additional file 13: Repeat content in HR-12, Nipponbare and 93–11 genomes. (XLSX 19 kb) Additional file 14: Chromosome-wise distribution of repeat elements in HR-12 genome. (XLSX 29 kb) Additional file 15: Functional annotation of variants in Co-39, HR-12, Tetep and Tadukan varieties. (XLSX 18 kb) Additional file 16: Number of effects of SNPs and InDels on various regions of genes. (XLSX 16 kb)

## Abbreviations

Ala: Alanine
AVR: Avirulence
bp: basepair
BWA: Burrows-Wheeler Aligner
cDNA: Complementary DNA
ESTs: Expressed sequence tags
GO: Gene ontology
InDels: Insertions and Deletions
Kb: Kilobase
LINEs: Long Interspersed Nuclear Elements
LRR: Leucine-rich repeat
LTR: Long terminal repeat
Mb: Million bases
MITEs: Miniature Inverted-repeat Transposable Elements
MP: Mate pair
NB-ARC: Nucleotide-binding adaptor shared by APAF-1 R proteins, and CED-4
nts: Nucleotides
PE: paired-end
Phe: Phenylalanine
PK: Protein kinase
PVC: Polyvinyl chloride
QTLs: Quantitative trait loci
R: Resistance
Ser: Serine
SINEs: Short Interspersed Nuclear Elements
SNPs: Single nucleotide polymorphisms
SSRs: Simple sequence repeats
Ts: Transition
Tv: Transversion
