# Supplementary figures and images for: Indica rice genome assembly, annotation and mining of blast disease resistance genes
Source: BMC Genomics. 2016 Mar 16;17:242. doi: 10.1186/s12864-016-2523-7 (PMC4793524; doi:10.1186/s12864-016-2523-7)

## Slide 1
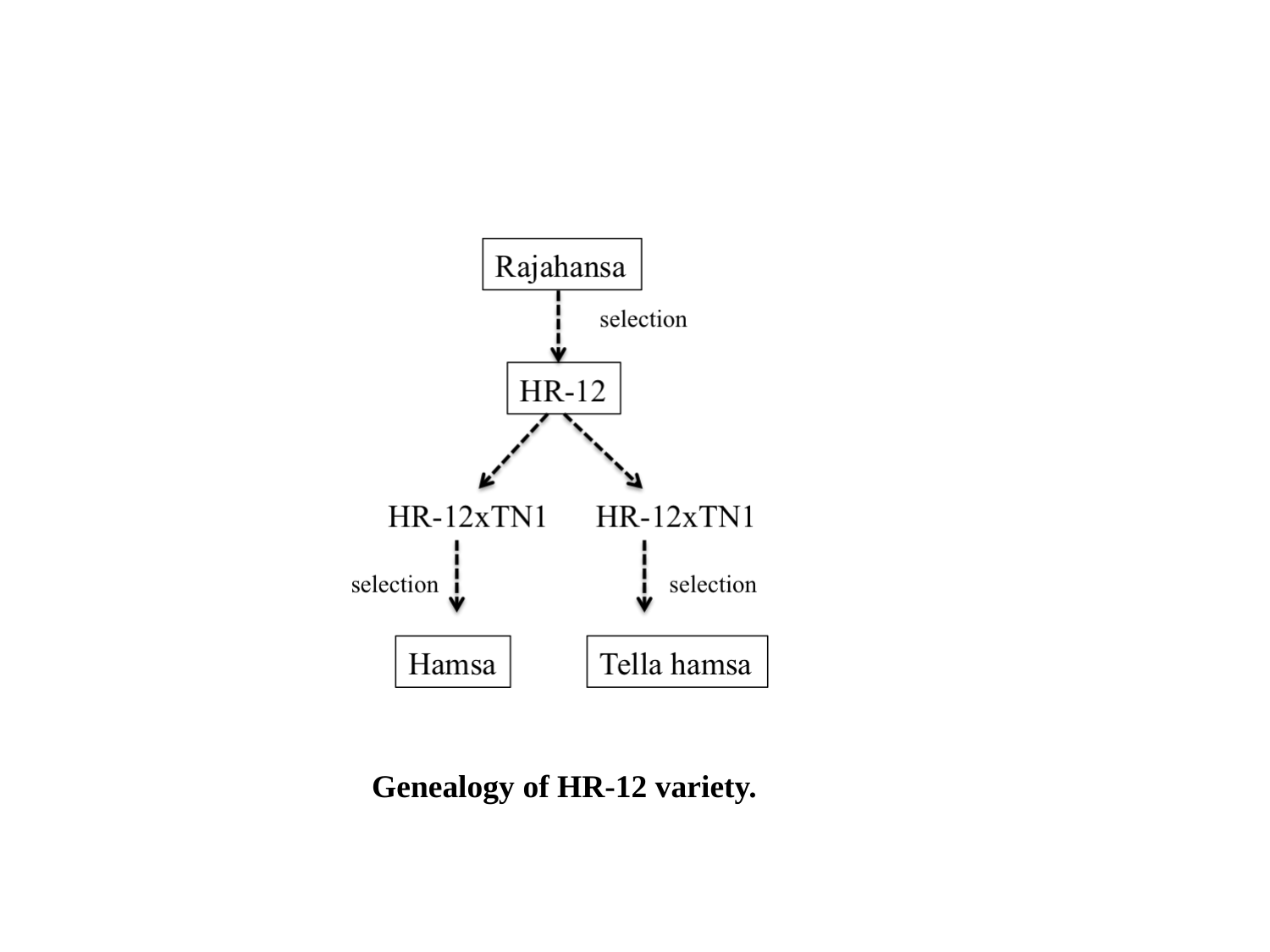

Genealogy of HR-12 variety.

Supplement: Additional file 1: — Genealogy of HR-12 variety. (PPTX 76 kb) [file 12864_2016_2523_MOESM1_ESM.pptx]
