# Supplementary material for: Indica rice genome assembly, annotation and mining of blast disease resistance genes
Source: BMC Genomics. 2016 Mar 16;17:242. doi: 10.1186/s12864-016-2523-7 (PMC4793524; doi:10.1186/s12864-016-2523-7)
Supplement: Additional file 3: — Analysis workflow followed for assembling Illumina and PacBio sequence reads of HR-12. (PPTX 129 kb) [file 12864_2016_2523_MOESM3_ESM.pptx]

## Slide 1
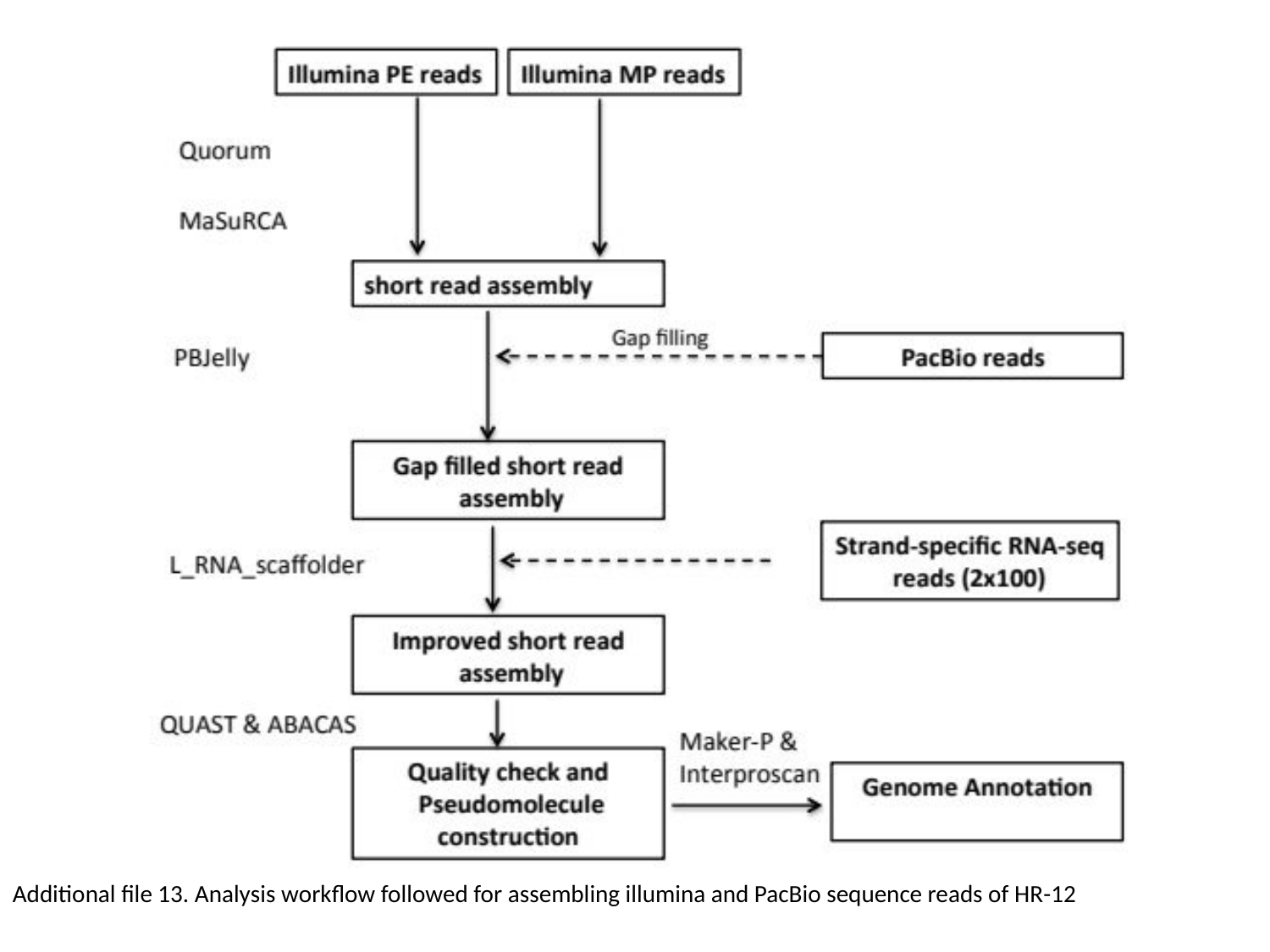

Additional file 13. Analysis workflow followed for assembling illumina and PacBio sequence reads of HR-12
