# Supplementary material for: Indica rice genome assembly, annotation and mining of blast disease resistance genes
Source: BMC Genomics. 2016 Mar 16;17:242. doi: 10.1186/s12864-016-2523-7 (PMC4793524; doi:10.1186/s12864-016-2523-7)
Supplement: Additional file 7: — Frequency histograms of numbers of nuclei per channel as a function of relative fluorescence in Pisum (internal standard), Co-39, HR-12, Tetep and Tadukan. The ‘x’ and ‘y’ axes represents number of nuclei and linear fluorescence, respectively. (PPTX 298 kb) [file 12864_2016_2523_MOESM7_ESM.pptx]

## Slide 1
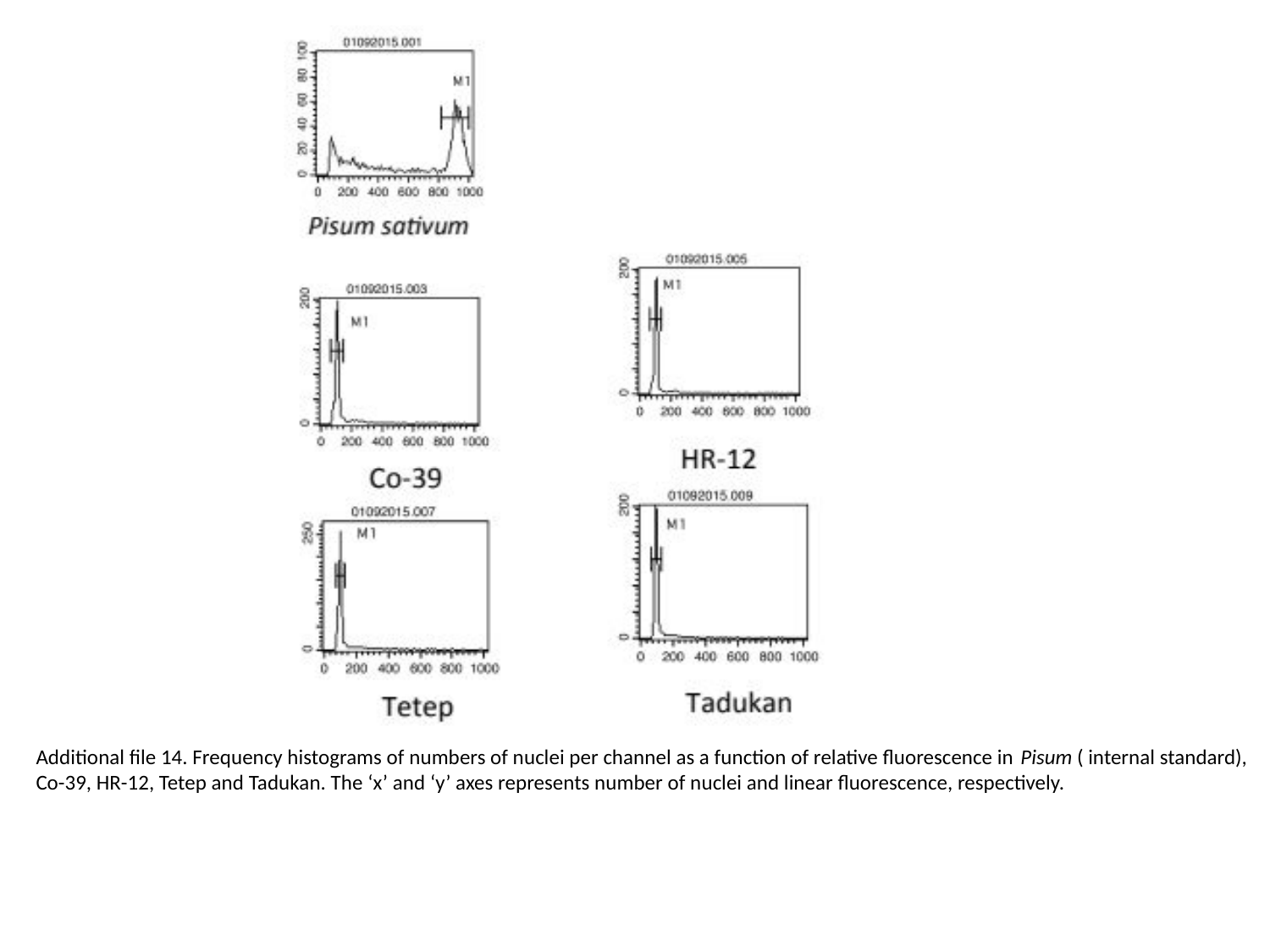

Additional file 14. Frequency histograms of numbers of nuclei per channel as a function of relative fluorescence in Pisum ( internal standard), Co-39, HR-12, Tetep and Tadukan. The ‘x’ and ‘y’ axes represents number of nuclei and linear fluorescence, respectively.
