# Supplementary material for: Indica rice genome assembly, annotation and mining of blast disease resistance genes
Source: BMC Genomics. 2016 Mar 16;17:242. doi: 10.1186/s12864-016-2523-7 (PMC4793524; doi:10.1186/s12864-016-2523-7)
Supplement: Additional file 8: — Comparison of syntenic blocks among HR-12, 93–11, and Nipponbare genomes. (a) syntenic blocks between HR-12 and 93–11. (b) syntenic blocks between HR-12 and Nipponbare and (c) syntenic blocks between HR-12, 93–11 and Nipponbare. (PPTX 3804 kb) [file 12864_2016_2523_MOESM8_ESM.pptx]

## Slide 1
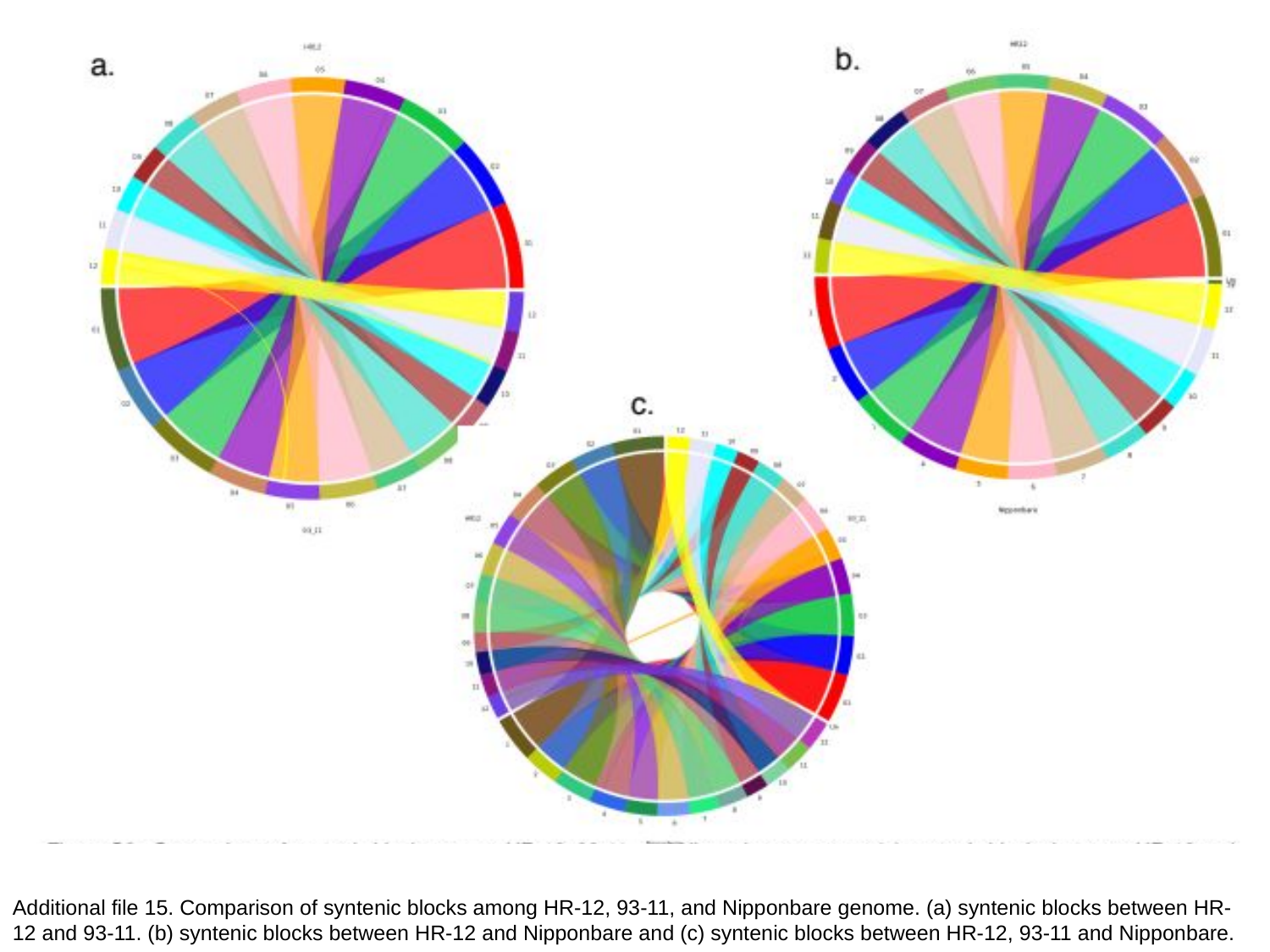

Additional file 15. Comparison of syntenic blocks among HR-12, 93-11, and Nipponbare genome. (a) syntenic blocks between HR-12 and 93-11. (b) syntenic blocks between HR-12 and Nipponbare and (c) syntenic blocks between HR-12, 93-11 and Nipponbare.
