# Supplementary material for: Indica rice genome assembly, annotation and mining of blast disease resistance genes
Source: BMC Genomics. 2016 Mar 16;17:242. doi: 10.1186/s12864-016-2523-7 (PMC4793524; doi:10.1186/s12864-016-2523-7)
Supplement: Additional file 11: — Number of SSRs in Illumina short and PacBio long read assemblies (a), Number of tri and tetra SSRs of HR-12, 93–11 and Nipponbare genomes (b). Distribution of tetra type SSRs in HR-12, Nipponbare and 93–11 genomes (c). (PPTX 183 kb) [file 12864_2016_2523_MOESM11_ESM.pptx]

## Slide 1
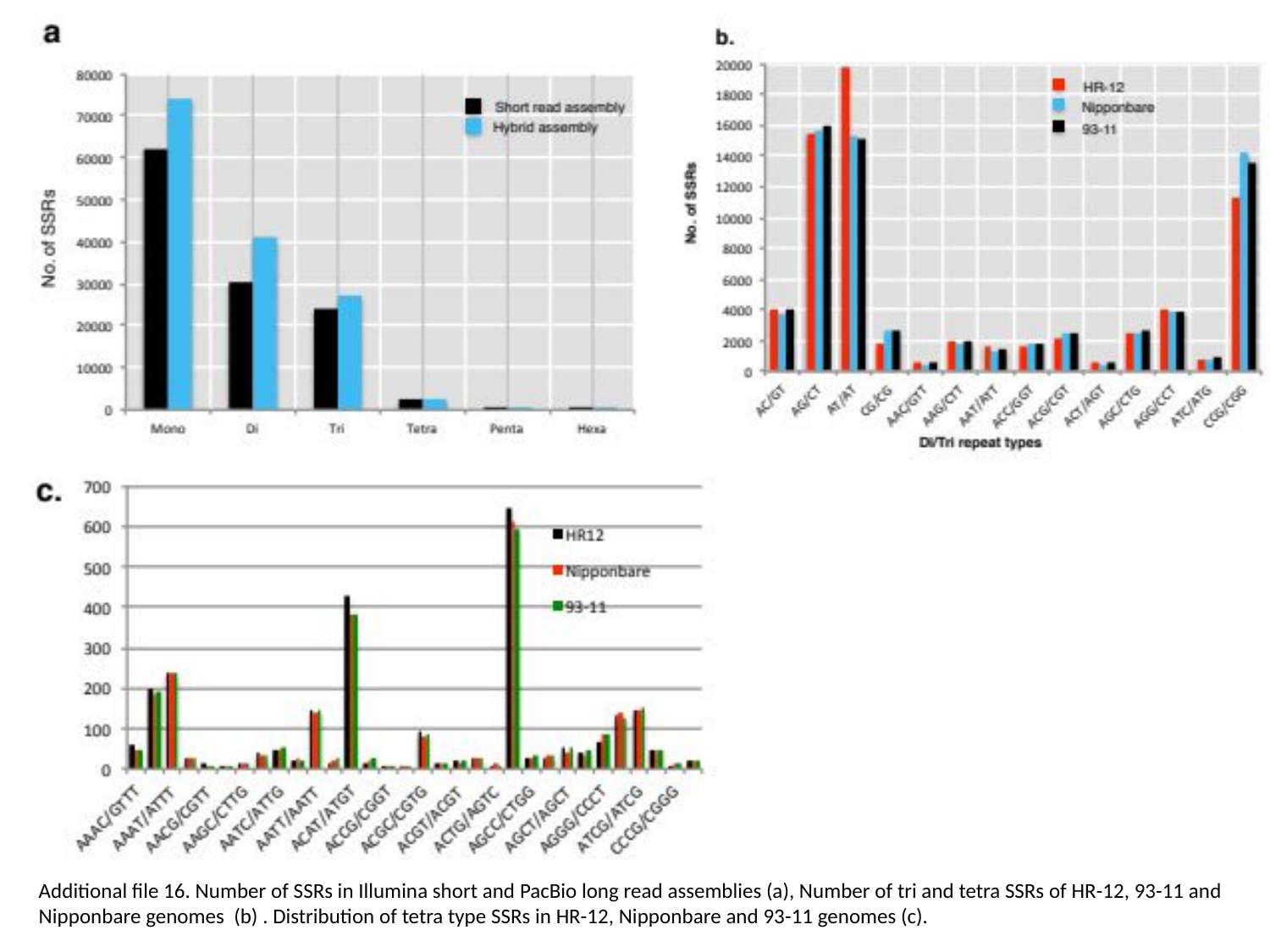

Additional file 16. Number of SSRs in Illumina short and PacBio long read assemblies (a), Number of tri and tetra SSRs of HR-12, 93-11 and Nipponbare genomes (b) . Distribution of tetra type SSRs in HR-12, Nipponbare and 93-11 genomes (c).
